# Examining the joint coordination during dynamic balance learning using vector coding and statistical parametric mapping analyses

**DOI:** 10.1038/s41598-023-44216-1

**Published:** 2024-01-19

**Authors:** Sungmin Kim, Feng Qu, Yi Wang, Wing-kai Lam

**Affiliations:** 1 Institute of School Physical Education, Korea National University of Education, Cheongju, Chungbuk Republic of Korea; 2 Biomechanics Laboratory, Beijing Sports University, Beijing, China; 3 Department of Physical Education, Renmin University of China, Beijing, China; 4 Sports and Social Development Research Center, Renmin University of China, Beijing, China; 5 Sports Information and External Affairs Centre, Hong Kong Sports Institutes, Sha Tin, Hong Kong China

**Keywords:** Risk factors, Health care, Disease prevention

## Abstract

We aimed to examine the changes in balance performance, kinematic variables, and joint coordination of the lower extremities during the Y-balance learning task. Twenty female university students completed five consecutive blocks of Y-balance learning from days 3 to 7 (135 trials). Pre-tests and tests were performed on days 1 and 9. Maximum reach distance, peak joint angle, and joint coordination in the anterior (AL), posterolateral (PL), and posteromedial (PM) directions were measured to determine the efficacy of Y-balance performance. A repeated measures ANOVA was performed for the maximum reach distance across learning blocks to confirm whether learning had occurred. Our results indicated that the maximum reach distance on day 5 was longer than that on other learning days. The maximum reach distance significantly increased in the PL and PM directions after learning. The hip flexion (PL/PM), abduction (PM), internal rotation (PM), and external rotation (PL) angles increased after learning. The knee joint flexion angle increased in both AL and PL directions. Only the ankle dorsiflexion angle increased in the AL direction. Joint coordination indicated that the knee and hip joints performed simultaneously during internal rotation. Ankle-knee joint coordination was performed using dorsiflexion and flexion strategies. Statistical parametric mapping analysis indicated significant differences in the ankle sagittal plane in the AL direction, hip horizontal and hip/knee sagittal planes in the PL direction, and hip/knee sagittal and hip frontal/horizontal planes in the PM direction. These data suggest that the dynamic balance ability of the novice participants improved in relation to changes in coordination patterns after learning. The results of this study can be applied to other populations to improve their dynamic balance and prevent fall injuries.

## Introduction

The Star Excursion Balance Test (SEBT) is a reliable tool for evaluating dynamic limits of stability and asymmetrical balance in the anterior, posteromedial, and posterolateral directions^1–3^. It can be used to develop better dynamic balance, improve athletic performance, promote rehabilitation sessions, and reduce the risk of fall injury^1,4^. To quantify SEBT, the Y-balance test (YBT) protocol was developed to continuously assess the eight directions of movement. The YBT is a functional test that measures strength, flexibility, neuromuscular control, balance, stability, and range of motion (ROM)^5–7^. It can provide a positive indicator for injury assessment in athletes^5,8^. Plisky et al.^1^ discovered a greater than 4 cm difference between the legs in the anterior reach direction, associated with a 2.5 times greater injury risk of acute lateral ankle sprain and chronic ankle instability.

Postural or balance control is the ability to maintain a base of support with minimal movement while maintaining a stable position^9^. To maintain the dynamic balance of a movement, the precise transmission of sensory information to mechanical receptors, including muscles, ligaments, and skin, is required^10^. However, inappropriate sensory signal transmission to mechanical receptors, including increased muscle contraction frequency and decreased joint position sense, may impair postural stability, resulting in a higher risk of ankle sprains, chronic ankle instability, and anterior cruciate ligament injury^1,11–17^. Thus, implementing efficient balance training programs is warranted^5^.

To maintain good dynamic balance, multiple joint movements are coordinated to control the various degrees of freedom at each joint in proper relations^18^ and integrate all motor units, muscles, segments, and joints into functional units for smooth and goal-directed movements^19^. Traditional approaches, such as angle–angle diagrams denoting the relationship between two joints on a graph, are used to analyze joint coordination^19^. However, quantifying the movements is challenging because they can only provide a qualitative expression for joint coordination. In contrast, using a vector coding method can provide a measure of continuous joint coupling to quantitatively describe the relationship between two segments or joints and indicate how one segment/joint influences another^20,21^. The vector coding approach calculates the relative excursions between successive sampled data points of an angle–angle diagram and the resultant angle (referenced to the horizontal) between these points to quantify the inter-joint coordination^22^.

Most previous studies on YBT have compared ankle instability and control groups and analyzed single joints in the sagittal plane of the lower extremity^18,23^. While explicit instructions are often used to facilitate YBT learning, how the dynamic balance of YBT can be coordinated and developed across the entire learning process is unknown. The objective of this study was to examine changes in the YBT performance, kinematic variables, and joint coordination of the lower extremities of novice learners throughout the learning process. In this study, the maximum reach distance, key kinematic variables, and joint coordination were evaluated to assess YBT learning and actual dynamic balance ability in both pre- and post-tests. The maximum reach distance during the learning phase was recorded to observe learning effects. Furthermore, statistical parametric mapping (SPM) was used to examine time-series kinematic data to identify the exact differences before and after balance learning. We hypothesized that the maximum reach distance would increase gradually during the learning phase and that the lower extremity joint coordination would change from a single joint to multi-joint coordination to achieve a longer maximum reach distance. Understanding the kinematics and coordination changes across the YBT could enhance performance optimization and identify injury risks to inform training programs for various populations.

## Methods

### Participants

Twenty female university students who were novices to the YBT were recruited for this study. Only female participants were recruited because they have lower strength and balance ability than male participants. Their average age, height, and weight were 22.0 ± 1.73 years, 162.3 ± 3.3 cm, and 49.9 ± 2.6 kg, respectively. None of the participants reported injuries to the lower-extremity joints or muscles in the past 6 months. Participants with a history of lower extremity, vestibular, visual, or balance impairment;, cardiovascular disease; or neurological disease were excluded from the study^24^. All participants were right-leg dominant, which was confirmed by asking them to kick a ball toward a forward target^8^. The experimental procedure was approved by the Ethics Committee of Beijing Sports University, and the study protocol was performed in accordance with the Declaration of Helsinki. Written informed consent was obtained from each participant before the start of the experiment.

### Apparatus and tasks

A standard YBT kit (Move 2 Perform; Evansville, IN, USA) was used to conduct the YBT. The balance kit had three cylindrical tubular moveable bars (reach indicator with marked increments of 0.5 cm) connected at the center of the balance kit. Each participant was instructed to maintain balance on their right leg (supporting leg) while simultaneously pushing the “reach indicator” as far as possible with the left leg (non-supporting leg) in the anterior, posterolateral, and posteromedial directions consecutively.

During the pre-learning block (day 1) and test blocks (day 9), the same experimenter placed 38 reflective markers over the bony landmarks of each participant to determine the kinematic variables of the lower extremity^25^. Motion-capturing systems with eight cameras (Qualisys, Gothenburg, Sweden; sampling at 200 Hz) were used during all pre-learning and test blocks to collect kinematic information of the lower extremities.

### Procedure

This study was designed with a pre-learning test block (day 1), learning blocks (days 3–7), and test blocks (day 9). The participants performed all the tests and learning at the biomechanical laboratory. On day 1, each participant was given an introductory session on the research procedure, requirements of the pre-learning, learning, and test blocks, and the YBT method. During the pre-learning test (day 1), the participants performed a 3-min self-directed warm-up and stretching^5^, followed by marker placement. For each practice trial, all participants were instructed to consecutively push the “reach indicator” with the non-supporting leg (left leg) in the anterior, posterolateral, and posteromedial directions while maintaining a single-leg stance (right leg). Five practice trials on Y balance were provided^23,26^. One-minute and 3-min rest periods were provided between the trials and blocks, respectively. Five successful trials for each reach direction were evaluated as the pre-learning baseline.

All participants rested on day 2. The Y-balance learning blocks were provided from days 3 to 7, with nine blocks of three trials (a total of 27 trials), with a 3-min rest separating each block on each learning day. The maximum pushing-reach distances were recorded in all three directions. All participants received the same instruction sheet with eight explicit instructions extracted from the American College of Sports Medicine guidebook and specific instructions described in previous studies^27–29^. Briefly, the participants were instructed as follows: (1) start performance after stabilizing the body during a single-leg stance; (2) support your toe, ankle, and knee joints as firmly as possible when your body goes down; (3) slowly perform and maintain balance during the task; (4) push the indicator box with the toe of the non-supporting leg; (5) imagine pushing the exact position during the posterior directions; (6) lean your upper body forward; (7) breathe out while pushing and breathe in during return; and (8) relax and maintain balance throughout. At the beginning of each learning block of the three trials, participants were reminded to use the given instructions to learn the YBT. No kinematic information was collected during the learning days.

**Figure 1:**
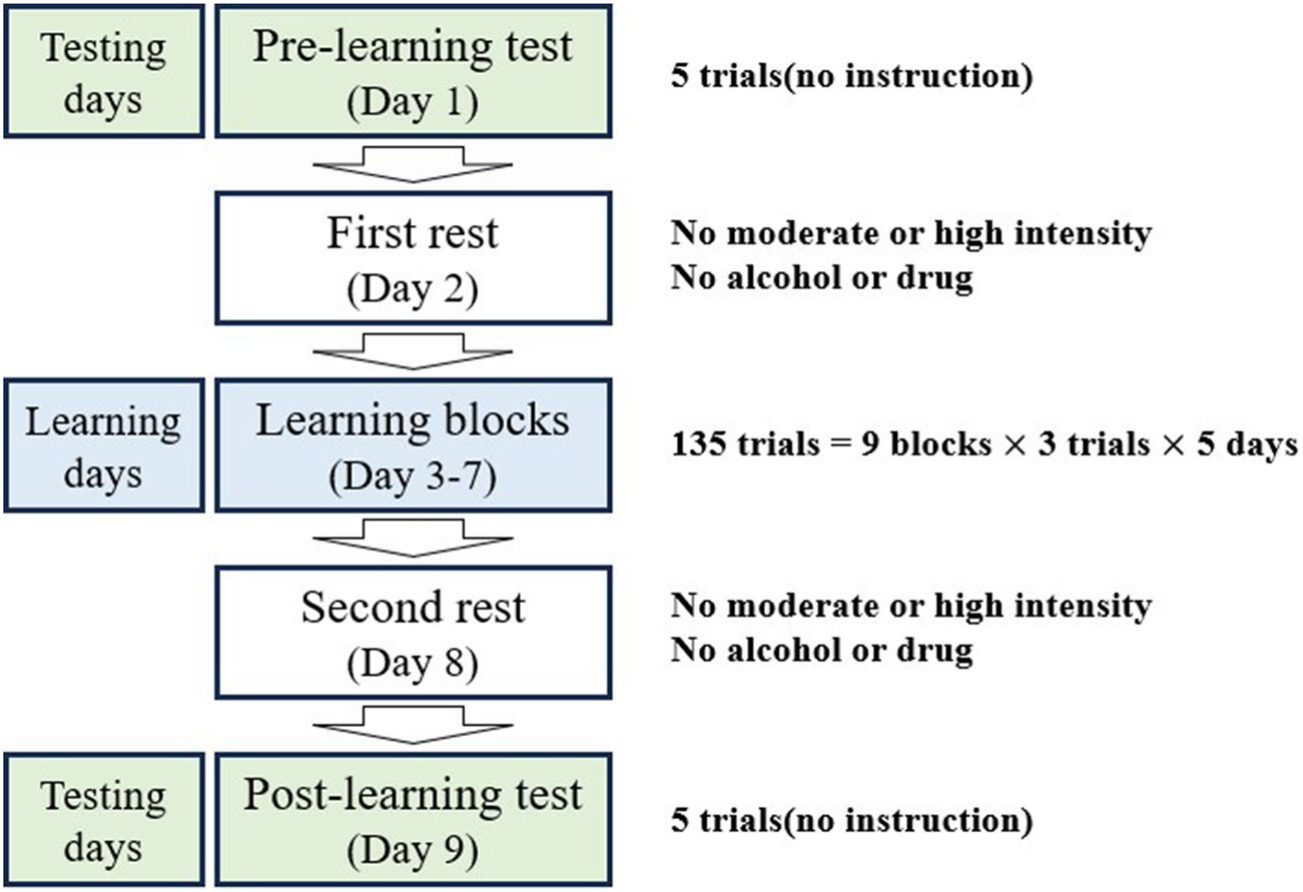
Study design.

After another resting day (day 8), the participants performed the same YBT in the test blocks on day 9. The experimental procedure and subject preparation (reflective markers for motion analysis measurements) were identical to those in the pre-learning phase. During the test blocks, all participants were not reminded of the instructions but were required to maximize their performance (Fig. 1).

### Dependent variables

**Figure 2:**
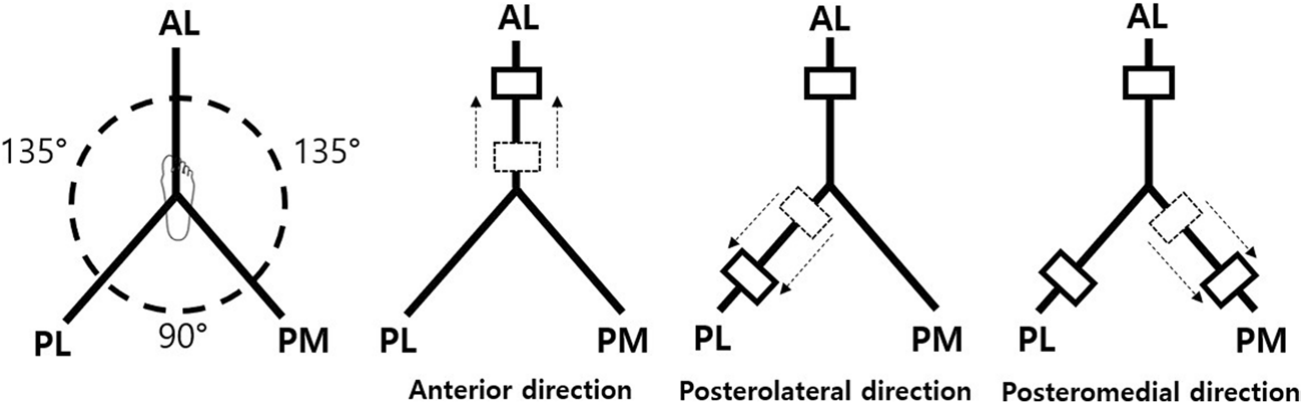
The definition of anterior, posteromedial, and posterolateral directions.

The phases of anterior, posteromedial, and posterolateral directions are defined in Fig. 2.

#### Maximum reach distance

The maximum reach distance was calculated as the absolute reach value normalized to individual limb length. The limb length of the participants was measured from the anterior superior iliac spine to the medial malleolus using tape measures. The normalized value was calculated as reach distance divided by limb length and multiplied by 100% to express reach distance as a percentage of dominant limb length.

#### Peak joint angle

The joint kinematic model was constructed based on the standing trial using Visual3D software (C-Motion Inc., Kingston, Canada). The joint angle was calculated as the orientation of the distal segment relative to the proximal segment based on the rigid body assumption. The Cardan sequence (x-y-z) was used to define the rotation of the segments in the local coordinate system. The joint center, segments, and their orientation from the proximal and distal endpoints were specified for further analyses. The calibration procedure ensured that + X represented hip flexion, knee extension, and ankle dorsiflexion, and − X represented hip extension, knee flexion, and ankle plantar flexion. + Y represented hip adduction, knee varus, and ankle inversion according to the right-hand thumb rules^30^.

#### Joint coordination

Vector coding was applied to quantify the coordination between the joints and provide interpretable data^21,31^. This technique quantifies the coordination patterns of any two pairs of joints (flexion–extension of the ankle-knee/flexion–extension of the knee-hip). The coupling angle was calculated using vector coding as the angle between the absolute resultant vector between two adjacent data points and its orientation to the right horizontal^21^. Joint coordination coupling angles (γ, from 0 to 360°) were derived from angle–angle plots based on the approach from previous studies^32^.

The bins were defined as four unique coordination patterns (in-phase, anti-phase, proximal phase, and distal phase), consistent with previous studies^32^. The in-phase strategy, denoted as the two joint couples, rotates simultaneously in the same direction; the anti-phase strategy, denoted as the two joint couples, rotates simultaneously in opposite directions; and the proximal- or distal-phase strategy is denoted as the coordination that occurs only in the proximal segment or only in the distal segment^33^. However, the knee joint angle data in the sagittal plane were extracted based on right-hand rules. In this study, the bins in the sagittal plane were interpreted inversely for the knee joint angle (see the sagittal plane in Table 1).

**Table 1:** Definition of the coupling angle on the thigh/shank/foot segment and coordination pattern based on the vector coding.

		Pattern	Coupling angle definitions
Sagittal plane		In-phase	112.5 ≤ $\gamma$≤157.5, 292.5 ≤ $\gamma$≤337.5
		Anti-phase	22.5 ≤ $\gamma$≤67.5, 202.5 ≤ $\gamma$≤247.5
		Proximal phase	0 ≤ $\gamma$≤22.5, 157.5 ≤ $\gamma$≤202.5, 337.5 ≤ $\gamma$≤360
		Distal phase	67.5 ≤ $\gamma$≤112.5, 247.5 ≤ $\gamma$≤292.5
Frontal plane		In-phase	22.5 ≤ $\gamma$≤67.5, 202.5 ≤ $\gamma$≤247.5
		Anti-phase	112.5 ≤ $\gamma$≤157.5, 292.5 ≤ $\gamma$≤337.5
		Proximal phase	0 ≤ $\gamma$≤22.5, 157.5 ≤ $\gamma$≤202.5, 337.5 ≤ $\gamma$≤360
		Distal phase	67.5 ≤ $\gamma$≤112.5, 247.5 ≤ $\gamma$≤292.5
Horizontal plane		In-phase	22.5 ≤ $\gamma$≤67.5, 202.5 ≤ $\gamma$≤247.5
		Anti-phase	112.5 ≤ $\gamma$≤157.5, 292.5 ≤ $\gamma$≤337.5
		Proximal phase	0 ≤ $\gamma$≤22.5, 157.5 ≤ $\gamma$≤202.5, 337.5 ≤ $\gamma$≤360
		Distal phase	67.5 ≤ $\gamma$≤112.5, 247.5 ≤ $\gamma$≤292.5

### Statistical analysis

All statistical analyses were performed using the SPSS statistical analysis computer program (SPSS 23.0, Inc., Chicago, IL, USA). All variables are expressed as mean ± standard deviation. An analysis of variance (ANOVA) with repeated measures was performed for the maximum reach distance data across all learning blocks, followed by Bonferroni-corrected post-hoc tests. To assess the effects of learning on balance performance and kinematics, paired t tests were used to compare the differences in joint angle and coupling angle variables between the pre- and post-learning tests. The alpha level was set at 0.05.

SPM was also used to statistically examine the time series for all kinematic data. The SPM analyses were conducted in MATLAB (MathWorks Inc., Natick, MA, USA) using the open-source software package spm1D 0.4^34^ (www.spm1d.org). Between-condition statistical analyses were conducted as described by Pataky^34^. Briefly, the null hypothesis was rejected if the experimentally computed t value between the pre- and post-test conditions for trajectory 1D data exceeded the critical value expected for smooth, 1D multivariate Gaussian data in an infinite number of experiments involving smooth 1D data. The mean trajectory data for the reaching direction of each leg were computed across participants and test blocks. The significance level was set at *p* < 0.05.

## Results

### Learning progress (days 3–7)—maximum reach distance

One-way ANOVA indicated significant main effects in the posterolateral and posteromedial directions during the learning phase (posterolateral: F = 61.286, *p* = 0.000, *η*^*2*^ = 0.763; posteromedial: F = 4.787, *p* = 0.018, *η*^*2*^ = 0.201) (Tables 2 and 3). Post-hoc analysis revealed that the maximum reach distance on day 5 was significantly longer than that on the other learning days (Table 2).

**Table 2:** Means and standard deviations for maximum reach distance across learning blocks (day 3 to day 7).

	Anterior	Posterolateral	Posteromedial
Day 3	57.27 ± 4.82	63.85 ± 9.54^a,b,c,d^	62.83 ± 9.61^c^
Day 4	58.37 ± 4.40	64.49 ± 9.36^a,e,f,g^	62.19 ± 8.96^e^
Day 5	58.04 ± 3.95	67.28 ± 9.28^b,e,f,i^	64.18 ± 9.11^e,h,i^
Day 6	57.93 ± 3.61	65.86 ± 9.03^c,f,h,j^	62.54 ± 7.94^c,h^
Day 7	58.70 ± 3.35	69.46 ± 8.11^d,g,i,j^	65.05 ± 7.48^i^
F	2.625	61.286	4.787
*p*	0.084	0.000*	0.018*
*η*^*2*^	0.121	0.763	0.201

^a^indicates a significant difference between days 3 and 4, ^b^indicates a significant difference between days 3 and 5, ^c^indicates a significant difference between days 3 and 6, ^d^indicates a significant difference between days 3 and 7, ^e^indicates a significant difference between days 4 and 5, ^f^indicates a significant difference between days 4 and 6, ^g^indicates a significant difference between days 4 and 7, ^h^indicates a significant difference between days 5 and 6, ^i^indicates a significant difference between days 5 and 7, ^j^indicates a significant difference between days 6 and 7.

*p* < .05.

Significant values are in bold.

**Table 3:** Comparisons of reach distance, time, and kinematic variables between pre- and post-test for ankle, knee, and hip joints.

	Pre-test			Post-test			t	*p*	95% Confidence Interval
	Anterior	Posterolateral	Posteromedial	Anterior	Posterolateral	Posteromedial			
Reach distance(%)	57.49 ± 4.70	63.78 ± 9.97^b^	63.00 ± 10.14^c^	58.24 ± 4.67	69.19 ± 8.83	68.04 ± 7.10	1) − 1.217	1) 0.238	1) − 2.04, 0.53
							2) − 3.632	2) 0.000*	2) − 8.53, − 2.29
							3) − 3.672	3) 0.000*	3) − 7.91, − 2.16
Time(s)	2.14 ± 0.72^a^	2.67 ± 0.75^b^	3.41 ± 0.99^c^	2.54 ± 0.91	3.36 ± 1.06	4.00 ± 1.31	1) − 2.611	1) 0.017*	1) − 0.72, − 0.08
							2) − 5.073	2) 0.000*	2) − 0.98, − 0.40
							3) − 2.781	3) 0.012*	3) − 0.98, − 0.14
Hip Joint (°)									
Adduction	2.28 ± 4.09	− 6.17 ± 6.23	0.56 ± 6.37	0.91 ± 4.00	− 7.13 ± 5.83	0.22 ± 5.15	1) 1.486	1) 0.154	1) − 0.55, 3.29
							2) 0.796	2) 0.436	2) − 1.55, 3.47
							3) 0.318	3) 0.754	3) − 1.88, 2.55
Abduction	14.96 ± 5.31	7.44 ± 8.45	4.20 ± 6.52^c^	15.06 ± 5.61	8.12 ± 7.38	14.10 ± 6.34	1) − 0.011	1) 0.913	1) − 1.95, 1.76
							2) − 0.473	2) 0.642	2) − 3.67, 2.32
							3) − 5.876	3) 0.000*	3) − 13.42, − 6.37
Flexion	38.19 ± 10.87	78.29 ± 7.21^b^	84.16 ± 10.37^c^	40.66 ± 16.12	92.39 ± 21.3	97.54 ± 21.20	1) − 0.949	1) 0.354	1) − 7.92, 2.97
							2) − 2.678	2) 0.015*	2) − 25.12, − 3.08
							3) − 2.660	3) 0.015*	3) − 23.92, − 2.85
Internal rotation	13.69 ± 11.51	10.96 ± 11.67	9.42 ± 12.10^c^	14.90 ± 10.87	13.82 ± 10.41	16.01 ± 7.12	1) − 0.365	1) 0.719	1) − 8.14, 5.72
							2) − 0.834	2) 0.415	2) − 10.04, 4.31
							3) − 2.329	3) 0.031*	3) − 12.51, − 0.66
External rotation	3.58 ± 11.35	0.44 ± 12.31^b^	− 6.59 ± 13.67	6.88 ± 12.20	− 10.38 ± 10.82	− 7.23 ± 14.10	1) − 0.995	1) 0.332	1) − 10.22, 3.63
							2) 2.955	2) 0.008*	2) 3.15, 18.49
							3) 0.165	3) .871	3) − 7.42, 8.68
Knee Joint (°)									
Varus	6.36 ± 7.52	7.30 ± 7.05	23.13 ± 11.69	7.23 ± 9.77	6.30 ± 8.91	23.72 ± 11.99	1) − 0.337	1) 0.740	1) − 6.26, 4.52
							2) 0.425	2) 0.675	2) − 3.93, 5.94
							3) − 0.182	3) 0.857	3) − 7.24, 6.08
Valgus	− 4.07 ± 4.64	− 3.74 ± 6.81	0.71 ± 5.00	− 2.89 ± 5.77	− 2.79 ± 6.61	2.19 ± 7.87	1) − 1.009	1) 0.325	1) − 3.62, 1.27
							2) − 0.589	2) 0.563	2) − 4.28, 2.40
							3) − 0.897	3) 0.381	3) − 4.93, 1.97
Flexion	− 71.30 ± 5.33^a^	− 72.09 ± 7.49^b^	− 62.08 ± 13.50	− 80.31 ± 13.07	− 82.22 ± 13.53	− 68.83 ± 39.26	1) 3.271	1) 0.004*	1) 3.24, 14.77
							2) 2.744	2) 0.013*	2) 2.40, 17.85
							3) − 0.948	3) 0.355	3) − 21.23,16.73
Internal rotation	3.01 ± 10.34	1.61 ± 10.10	− 3.89 ± 9.63^c^	4.25 ± 9.74	5.17 ± 12.96	8.59 ± 8.62	1) − 0.586	1) 0.565	1) − 5.67, 3.19
							2) − 1.376	2) 0.185	2) − 8.98, 1.85
							3) − 4.184	3) 0.001*	3) − 18.74, 6.24
External rotation	− 7.00 ± 10.73	− 0.99 ± 6.63^b^	− 18.61 ± 10.70	− 7.78 ± 8.97	− 9.79 ± 8.98	− 17.64 ± 10.56	1) 0.387	1) 0.703	1) − 3.44, 5.00
							2) 3.582	2) 0.002*	2) 3.65, 13.93
							3) − 0.466	3) 0.646	3) − 5.34, 3.39
Ankle joint (°)									
Inversion	8.64 ± 5.67	5.16 ± 4.13	5.77 ± 4.38	8.90 ± 5.41	6.48 ± 6.09	5.52 ± 6.16	1) − 0.144	1) 0.887	1) − 4.00, 3.48
							2) − 1.057	2) 0.304	2) − 3.92, 1.28
							3) 0.197	3) 0.846	3) − 2.44, 2.95
Eversion	− 1.59 ± 3.74	− 2.95 ± 4.18	− 2.82 ± 4.69	0.39 ± 11.36	− 0.34 ± 11.18	− 1.23 ± 11.02	1) − 0.782	1) 0.444	1) − 7.28, 3.31
							2) − 1.052	2) 0.306	2) − 7.80, 2.58
							3) − 0.598	3) 0.557	3) − 7.13, 3.96
Dorsiflexion	22.94 ± 8.72^a^	29.08 ± 5.17	25.54 ± 6.41	34.20 ± 6.64	29.97 ± 6.93	28.58 ± 6.64	1) − 4.175	1) 0.023*	1) − 16.90, − 5.61
							2) − 0.760	2) 0**.**135	2) − 3.35, 1.56
							3) − 0.571	3) 0.669	3) − 5.50, − 0.56
Abduction	6.80 ± 9.19	3.35 ± 9.24	4.15 ± 10.31	9.27 ± 5.16	6.75 ± 5.49	5.26 ± 4.84	1) − 1.197	1) 0.246	1) − 6.77, 1.84
							2) − 1.553	2) 0.137	2) − 7.96, 1.17
							3) − 0.492	3) 0.628	3) − 5.83, 3.61
Adduction	− 3.95 ± 9.25	− 7.23 ± 9.42	− 7.06 ± 10.71	− 1.53 ± 4.12	− 3.75 ± 6.13	− 6.40 ± 5.50	1) − 1.176	1) 0.254	1) − 6.72, 1.88
							2) − 1.615	2) 0.123	2) − 8.00, 1.03
							3) − 0.288	3) 0.777	3) − 5.43, 4.12

1) the paired t test result of the anterior direction phase between pre- and post-test.

2) the paired t test result of the posterolateral direction phase between pre- and post-test.

3) the paired t test result of the posteromedial direction phase between pre- and post-test.

^a^ indicates a significant difference between pre- and post-test in the anterior direction phase,

^b^ indicates a significant difference between pre- and post-test in the posterolateral direction phase,

^c^ indicates a significant difference between pre- and post-test in the posteromedial direction phase.

* p < 0.05.

Significant values are in bold.

### Learning effectiveness (pre-test vs. post-test)

#### Maximum reach distance and performance time

The maximum reach distance in each direction was significantly longer in the post-test compared with the pre-test (posterolateral: t = − 3.632, *p* = 0.000; posteromedial: t = − 3.672, *p* = 0.000) (Table 4). Significant differences were observed in performance time between the pre- and post-YBT for each reaching direction (anterior: t = − 2.611, *p* = 0.017; posterolateral: t = − 5.073, *p* < 0.001; posteromedial: t = − 2.781, *p* = 0.012).

**Table 4:** Comparison of coupling angle between pre- and post-test.

	Pre-test			Post-test			t	*P*	95% Confidence Interval
	Anterior	Posterolateral	Posteromedial	Anterior	Posterolateral	Posteromedial			
AK									
Sagittal	166.17(S)^a^ ± 27.96^a^	168.13(S) ± 29.01	134.63(I) ± 16.35	135.43(I) ± 20.90^a^	167.74(S) ± 30.13	133.13(I) ± 16.30	1) 3.777	1) 0.001*	1) 13.70, 47.77
							2) 0.046	2) 0.964	2) − 17.23,18.00
							3) 0.331	3) 0.744	3) − 7.93, 10.92
Frontal	276.04(F) ± 28.47	274.66(F) ± 22.27	258.28(F) ± 27.30	277.51(F) ± 12.35	276.18(F) ± 12.60	269.02(F) ± 13.32	1) − 0.237	1) 0.815	1) − 14.45,11.51
							2) − 0.302	2) 0.766	2) − 12.01, 8.98
							3)− 2.028	3) 0.060	3) − 21.30, − 0.17
Horizontal	196.10(S) ± 27.86	190.50(S) ± 29.02	192.95(S) ± 21.21	183.65(S) ± 22.22	182.71(S) ± 21.99	190.26(S) ± 18.27	1) 1.838	1) 0.082	1) − 1.72, 26.64
							2) 1.034	2) 0.314	2) − 7.99, 23.58
							3) 0.472	3) 0.643	3) − 9.26, 14.65
KH									
Sagittal	81.85(S) ± 17.29	179.25(T) ± 23.46	168.43(T) ± 26.70	98.04(S) ± 38.83	184.53(T) ± 28.42	174.35(T) ± 33.31	1)− 1.690	1) .107	1) − 36.22, 3.86
							2) − 0.723	2) 0.478	2) − 0.55, 0.00
							3) − 0.822	3) 0.422	3) − 20.99, 9.15
Frontal	171.57(T) ± 11.16	140.26(A) ± 12.09	132.74(A) ± 14.31	168.49(T) ± 11.12	135.72(A) ± 8.98	130.26(A) ± 6.90	1) 1.293	1) 0.211	1) − 1.89, 8.04
							2) 1.333	2) 0.198	2) − 2.59, 11.68
							3) 0.792	3) 0.438	3) − 4.05, 9.00
Horizontal	184.00(T) ± 14.71	182.06(T) ± 16.10	270.16(S)^c^ ± 16.72	183.22(T) ± 12.98	181.90(T) ± 12.07	223.02(I) ± 12.89^c^	1) 0.225	1) 0.824	1) − 6.47, 8.04
							2) 0.046	2) 0.964	2) − 7.39, 7.72
							3)10.506	3) 0.000*	3) 37.75, 56.53

AK, Ankle-Knee; KH, Knee-Hip.

I, In-phase; A, Anti-phase; F, Foot-phase; S, Shank-phase; T, Thigh-phase.

1) the paired t test result of the anterior direction phase between pre- and post-test,

2) the paired t test result of the posterolateral direction phase between pre- and post-test,

3) the paired t test result of the posteromedial direction phase between pre- and post-test,

^a^indicates a significant difference between pre- and post-test in the anterior direction phase,

^b^indicates a significant difference between pre- and post-test in the posterolateral direction phase,

^c^indicates a significant difference between pre- and post-test in the posteromedial direction phase.

*p < 0.05.

Significant values are in bold.

#### Peak joint angle

**Figure 3:**
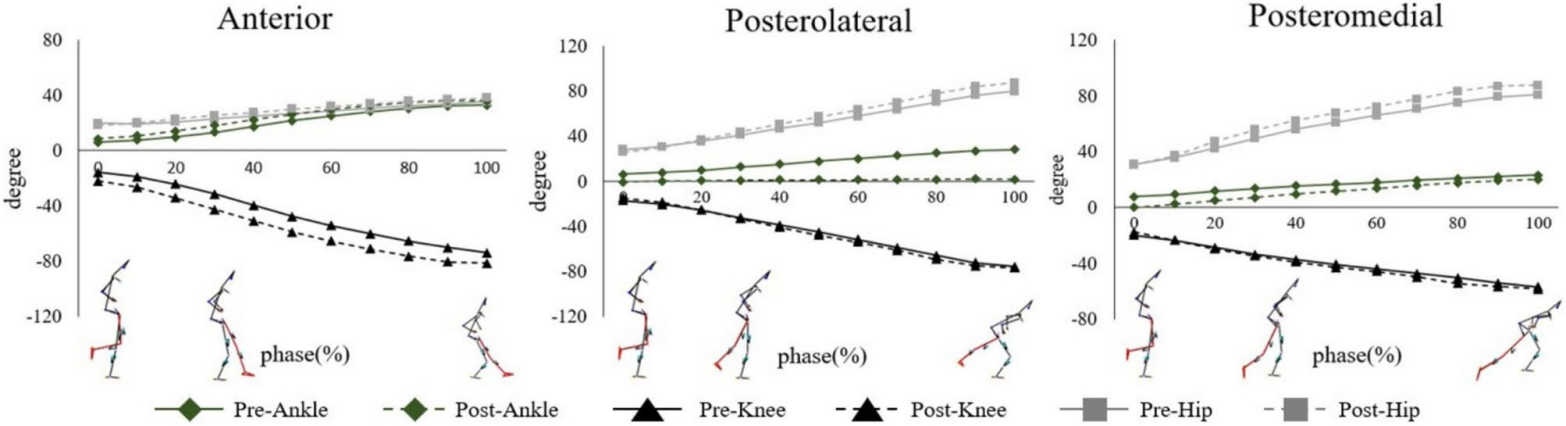
Change of each joint angle during the Y-balance task.

Significant differences were observed between the pre- and post-tests (Table 3 and Fig. 3). At the hip joint, the abduction angle in the pre-test significantly differed from that in the post-test in the posteromedial direction (t = − 5.876, *p* = 0.000). The peak hip flexion angle and ROM were significantly larger in the pre-test in the posteromedial (t = − 2.678, *p* = 0.015) and posteromedial (t = − 2.660, *p* = 0.015) directions than in the post-test. In the horizontal plane, the peak hip rotation angles were larger in the post-test in both the posteromedial (external rotation: t = 2.955, *p* = 0.008) and posteromedial (internal rotation: t = − 2.329, *p* = 0.031) directions.

Significant differences were observed between the sagittal and horizontal planes of the knee joints. The peak knee sagittal and horizontal angles and the ROMs increased post-test (knee flexion in the posterolateral: t = 3.271, *p* = 0.004 / posteromedial: t = 2.744, *p* = 0.0.013; internal rotation in the posteromedial: t = − 4.184, *p* = 0.001; external rotation in the posterolateral: t = − 3.582, *p* = 0.002). At the ankle joint, the peak dorsiflexion angle and ankle ROM in the anterior direction increased in the pre-test than in the post-test (t = − 4.175, *p* = 0.023).

#### *Coupling angle (*Table 4*)*

For ankle-knee coupling angles, participants exhibited a shank phase (knee flexion) in the anterior direction in the pre-test. However, in the post-test, the participants performed the in-phase strategy with ankle dorsiflexion and knee flexion simultaneously (t = 3.777, *p* = 0.001). A significant difference was observed in the knee-hip coupling angles in the posteromedial direction between the pre- and post-tests. All participants displayed a shank phase (knee internal rotation) at the pre-test. An in-phase strategy with knee and hip internal rotation was utilized simultaneously at the post-test in the posteromedial direction (t = 10.506, *p* < 0.001).

#### SPM (Statistical parametric mapping)

**Figure 4:**
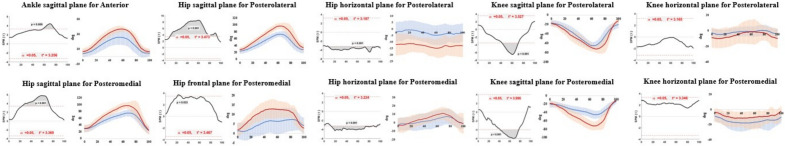
The mean differences of the joint between pre- and post-test. SPM greater than t* indicates a significant difference between pre- and post-test.

The SPM analysis with paired t test revealed significant differences between pre-test and post-test conditions during the movement phase (Fig. 4). Significant differences were observed at approximately 57–78% of the sagittal ankle plane in the anterior direction (*p* = 0.009). In the posterolateral direction, a significant difference was observed in hip horizontal (*p* < 0.001, 9–100%) and hip/knee sagittal planes (hip: *p* < 0.001, 9–93%; knee: *p* < 0.001, 42–71%). Hip/knee sagittal (hip: *p* < 0.001, 23–74%; knee: *p* < 0.001, 41–77%) and hip frontal/horizontal (frontal: *p* = 0.033, 20–27%; horizontal: *p* < 0.001, 12–94%) planes in the posteromedial direction were significantly greater compared with the pre-test.

## Discussion

This study examined biomechanical changes throughout the YBT learning process, as indicated by the analyses of maximum reach distance, kinematic and joint coordination of the lower extremities, and SPM in female novice adults. The findings of the maximum reach distance and joint angle across the learning days confirmed that all participants exhibited larger joint angles and performed multi-joint coordination in the three movement planes to achieve longer pushing distances. Our results also revealed different coordination strategies across the three lower-limb joints of the supporting leg.

During the learning phase, the maximum reach distance increased only in the posterolateral and posteromedial directions but not in the anterior direction, consistent with our hypothesis. This suggests that participants may rely on feed-forward control strategies to perform complex coordination for the maximum reach distance^16^. Such feed-forward control can predict future signs and achieve goals using various strategies based on information acquired through extensive practice^35,36^. Participants may respond to postural instability during the YBT by maintaining balance using various control strategies^16,37^. Moreover, the results of this study revealed a similar degree of change to that observed in a previous study^38^. A previous study^16^ suggested that performance in the anterior direction might not demonstrate a significant difference between test conditions, unlike in the posteromedial and posterolateral directions. One plausible explanation for this is that our participants were more likely to receive visual feedback when executing the reaching block position in the anterior direction.

Analysis of ankle-knee coupling angles confirmed that our participants changed the control strategy from a shank-phase (knee flexion) strategy in the sagittal plane in the anterior reaching direction to an in-phase strategy (ankle dorsiflexion and knee flexion simultaneously) during the post-test. All participants displayed a shank phase (knee internal rotation) in the horizontal plane during the pre-test, and an in-phase (knee and hip internal rotation simultaneously) was applied during the post-test. Moreover, larger hip flexion and external rotation angles were observed in the post-test than in the pre-test. These results confirm our hypothesis that the single-joint coordination strategy changed to multi-joint coordination after the learning blocks. Kang et al.^39^ reported significant differences in the posterior reach distance and sagittal plane hip angles. The correlation test results also suggested that the maximum reach distance was associated with the hip flexion angle and that the hip flexion angle could be the best predictor of posteromedial and posterolateral reach distances^39^. Another study reported smaller hip flexion in female athletes who underwent anterior cruciate ligament reconstruction than that in a healthy group^40^. Thus, an increased hip flexion angle after Y-balance learning may strongly influence reach distance in both posterior directions. Furthermore, abduction and internal rotation increased compared with those in the pre-test in posteromedial direction. All these changes provide insights into how movement strategies develop across learning for training and assessment reference. Considering that postural instability characteristics during single-leg support tend to exhibit a high COM position, the frequency of postural adjustments increases, and the COM leans backward and is lowered to maintain dynamic balance^41^. Simultaneously, the distance between the COM and the foot of the non-supporting leg gradually increases, and the maximum reach distance can be increased through external rotation of the hip joint. Therefore, all participants in our study exhibited greater hip flexion and could stretch far using the non-supporting leg through external rotation to achieve a larger maximum reach distance in the YBT. Our SPM analysis can help identify the exact differences throughout the movement phase. Significant differences existed in the posterolateral (9–100%) and posteromedial (12–94%) directions compared with the pre-learning phase. Horizontal plane movement causes body instability during single-leg stance, and the trunk and pelvis are twisted for leg-reaching movements in the posteromedial and posterolateral directions^26,27^. In this study, despite the increase in hip ROM in the horizontal plane, participants demonstrated improved postural control when adopting an unstable posture after learning, suggesting that the increase in postural control ability in the horizontal plane was associated with larger hip flexion and lower COM.

At the hip joint level, the participants increased their external hip rotation after learning. Further analysis of time-series data suggests that hip movement is mainly involved in the frontal (abduction) and horizontal (internal rotation) planes, which confirms that an increase in maximum reach distance is associated with a larger hip internal rotation movement after learning. As the posterolateral direction moves diagonally backward to the non-supporting leg (left leg), the trunk must tilt diagonally forward to maintain a base of support with minimal movement^1,42^. However, in the posteromedial direction, abduction and internal rotations increased after Y-balance learning, as the posteromedial direction requires increased hip ROM in the horizontal plane when pushing the block toward the medial side of the non-supporting leg^43^.

At the knee joint level, our data revealed a larger knee joint flexion angle in the anterior direction after Y-balance learning, consistent with the findings of previous studies in which participants increased knee flexion by 10° during post-test^44,45^. While a higher COM position may lead to postural instability, the height of the COM at the level of the knee flexion angle results in greater difficulty in maintaining balance in the posterolateral and posteromedial directions. Complex trunk movements in the diagonal direction must be controlled to increase reach distances in the posterolateral and posteromedial directions^46^. Hence, our participants could maintain body stability by shifting from the hip flexion and external rotation strategies toward the knee bending strategy in the posterolateral direction after 5 days of learning.

At the ankle joint level, the ankle joint increased dorsiflexion in the anterior direction after Y-balance learning. These results are supported by Hoch et al.^47^, who reported that ankle dorsiflexion is highly correlated with the anterior direction. The authors conducted 2 weeks of training for patients with chronic ankle instability, and the maximum reach distance improved in the anterior direction. This can be used as a clinical consideration for evaluating ankle dorsiflexion^43,47^.

The YBT is a complex task that requires precise endpoint movement control accomplished by multi-joint coordination. Our results revealed that internal knee rotation was performed only during the pre-test, and knee and hip internal rotations were performed simultaneously during the post-test. In the study by Koshino et al.^48^, the hip joint was also subjected to internal knee rotation when performing rotational movements such as cutting. Shank and femur internal rotation may shift the knee medially during knee flexion, resulting in hip adduction to a lower COM^49^. In another study by Overmoyer and Reiser^23^, knee flexion did not increase the maximum reach distance in the anterior direction, and the ankle performed knee flexion simultaneously. In our study, the participants demonstrated improvement in maximum reach distances by larger knee flexion and ankle dorsiflexion angles after 5 days of learning.

While verbal-motor instructions are commonly provided to learners to acquire new motor tasks, we provided instructions to our novice female participants to provide a cognitive representation of a motor task and elicit changes in movement execution for better Y-balance performance outcomes. Postural control is affected by both internal and external factors^50^. By reminding the participants with verbal instructions during the learning blocks, they can focus on predicting an unstable dynamic balance and minimize factors that interfere with the body’s equilibrium using various control strategies^51^. As such, our participants continuously modified their posture through the given instructions across the five learning days to achieve movement automaticity, resulting in changes in the kinematic variables and control strategies^52^. However, our participants may have made movement errors if excess explicit information was provided to learners^53^. Future studies should examine the effect of the extent of instructions on the changes in kinematic and control strategies throughout Y-balance learning.

Some limitations should be considered in interpreting our results. First, only short-term learning performance was reported, as it was more effective in providing initial insights. An extended learning and biomechanical analysis period should also be considered when determining the automatic stage. Second, young female novices were recruited in this study. Therefore, our results may not be generalizable to children or older populations. Future studies should examine the efficacy and control strategies for patients with chronic ankle instability or older populations to improve dynamic balance and prevent fall injuries.

## Conclusion

Learning of the YBT was accomplished, as indicated by the gradually increasing maximum reach distance and performance time across the learning blocks. After learning the YBT, larger dorsiflexion and knee flexion were observed in the anterior direction, while larger external hip and knee rotations were observed in the posterolateral direction and internal rotation in the posteromedial direction. Joint coordination revealed that the knee and hip joints performed simultaneously during internal rotation, and ankle dorsiflexion and knee flexion coordination were used. These results indicate that increased maximum reach could be associated with more harmonious multiple lower-extremity joint coordination.

## Data Availability

The datasets generated during and/or analyzed during the current study are available from the corresponding author on reasonable request.
